# The effect of aromatherapy on pain in individuals with diabetes: a systematic review and meta-analysis

**DOI:** 10.7586/jkbns.24.012

**Published:** 2024-05-24

**Authors:** Mi-Kyoung Cho, Mi Young Kim

**Affiliations:** 1 Department of Nursing Science, Chungbuk National University, Cheongju, Korea; 2 College of Nursing, Hanyang University, Seoul, Korea

**Keywords:** Pain, Diabetes mellitus, Aromatherapy, Meta-analysis

## Abstract

**Purpose:**

This study systematically analyzed the impact of aromatherapy on pain in individuals with diabetes.

**Methods:**

A search was performed in seven electronic databases based on the PICO-SD (Population, Intervention, Comparison, Outcome, Study Design) framework. The population (P) of interest was individuals with diabetes, and the intervention (I) included aromatherapy targeting pain reduction. The comparison (C) consisted of control groups that received no intervention, another intervention, or usual care. The outcome (O) measured was pain. The quality of the selected literature was assessed using the Joanna Briggs Institute checklist. In MIX 2.0 Pro, the pooled overall effect of pain was calculated using Hedge's g and a random-effects model, and heterogeneity was calculated using the Q statistic and Higgin's I2 values. Meta-regression and exclusion sensitivity analyses were performed.

**Results:**

Five articles and seven studies were included, showing a significant pooled overall effect of aromatherapy on diabetes-related pain (Hedge’s g = −1.83, 95% CI: −2.76 to −0.91). Meta-regression demonstrated that effectiveness in reducing pain was associated with studies conducted in West Asia, those with IRB approval, and those receiving funding. Additionally, interventions involving subjects under 60, lavender oil (vs. turpentine oil or blended oils), massage therapy (vs. topical application), fewer hours per session, and more repeated measurements (vs. pre/post measurements) were associated with pain reduction.

**Conclusion:**

Aromatherapy, especially with lavender oil, effectively manages diabetes-related pain. Short-duration massage application is also effective. A personalized selection of oil type and application method could optimize therapeutic outcomes for individuals with diabetes.

## INTRODUCTION

Complications experienced by diabetes often involve the nervous system, leading to severe and persistent pain that is challenging to manage. Neuropathic pain is commonly defined as pain resulting from a lesion of the somatosensory system, which results in faulty pain signaling [1]. Neuropathic pain is linked with various conditions such as spinal cord compression, HIV, amputation, fibromyalgia, multiple sclerosis, postherpetic neuralgia, and diabetic neuropathy. The pathogenesis of neuropathic pain in each of these conditions is contingent upon the underlying disease process. In diabetic neuropathy, prolonged hyperglycemia triggers numerous metabolic pathways, including the generation of free radicals and the accumulation of advanced glycation end products in the microvasculature supplying peripheral nerves [2]. These processes lead to direct toxicity, reduced blood flow, and subsequent degeneration of nerve fibers, along with heightened excitability of primary afferent nociceptors [2].

Research indicates that diabetic peripheral neuropathy affects a significant portion of diabetes, ranging from 16% to 26% [3]. This condition not only restricts physical activity but also impairs quality of life. Despite the utilization of various medication treatments including anticonvulsants, antidepressants, and opioids, neuropathic pain continues to pose a persistent challenge for diabetes [4]. The primary focus in addressing neuropathic pain revolves around symptom management. Considered a chronic condition, neuropathic pain often persists despite efforts to reverse the underlying cause. Managing this pain typically requires a multimodal approach, incorporating both pharmacological and nonpharmacological interventions [5]. Therefore, developing a comprehensive pain management plan involving non-drug methods becomes imperative. These may encompass massage therapy, acupuncture, transcutaneous electrical nerve stimulation units, trigger point injections, and spinal cord stimulators. Furthermore, there is growing interest in natural alternative options, such as essential oils, herbal remedies, and dietary supplements, especially in regions with a rich history of traditional medicine like Asia, Africa, and Latin America [6].

Numerous complementary and alternative therapies, including aromatherapy, are available for consideration. Aroma oils are favored due to their established safety for the human body [7]. Various oils, such as bergamot, cedarwood, Roman chamomile, geranium, ginger, jasmine, lavender, lemon, and tea tree, are commonly utilized in aromatherapy [8]. Furthermore, there are diverse methods for applying aromatherapy. Essential oils offer versatile application methods, including inhalation, topical application, or ingestion, with inhalation being the most prevalent. The majority of essential oils are deemed safe and have gained Food and Drug Administration (FDA) approval as ingredients in food and fragrances, being classified as "generally recognized as safe". However, it's worth noting that aromatherapy products typically do not require FDA approval unless they make specific claims regarding the treatment of diseases [8].

When aroma oil is inhaled through the mouth and nose, its scent particles interact with the limbic system of the brain via the skin, lungs, and circulatory system, resulting in rapid effects. This method proves effective in promoting mental and physical stability, mood enhancement, and improved mental concentration, as evidenced in existing literature [9]. Given the diverse array of oils, application methods, and timing considerations, determining the effectiveness of aromatherapy in pain management, identifying the most effective aroma oils, and establishing optimal application techniques pose challenges. Thus, it is crucial to conduct systematic research to ascertain the most suitable approach for alleviating pain in diabetes.

Therefore, in this study, with the research question 'Can aromatherapy reduce pain in diabetes?', a systematic literature review and meta-analysis were conducted to determine the effect of aromatherapy on reducing pain in diabetes, thereby providing the evidence for complementary and alternative therapy.

## MATERIALS AND METHOD

### Search strategy and data sources

For the search, two researchers searched a total of seven electronic databases or electronic journals (PubMed, Cochrane, EMBASE-OVID, CINAHL, World of Science, SCOPUS and RISS) for papers published in English and Korean until December 31, 2023. The search was conducted from January 18, 2024 to February 22, 2024, and the search strategy based on PICO-SD (Population, Intervention, Comparison, Outcome, Study Design) was shown in Table 1.

### Inclusion and exclusion criteria

The results of this study were reported with reference to the PRISMA 2020 checklist (accessed at https://www.prisma-statement.org/ on April 1, 2024). The selection criteria were as follows. The study population (P) was diabetes aged 19 years and older, the intervention (I) was aromatherapy, the control (C) was conventional care or no intervention, and the outcome (O) was pain. When two or more interventions were used in an article, each intervention was separated into its own study and the effect size was calculated. Only studies that presented the number of subjects, mean, and standard deviation were selected for accurate effect size calculation. The study design (SD) included randomized controlled trials (RCTs) and a quasi-experimental study. The exclusions were as follows: studies that the subjects were not adult diabetics; studies that the intervention was not aromatherapy; studies that pain was not reported as an outcome variable; studies that the mean, standard deviation, or number of subjects was not reported; studies not reported in English or Korean; studies that the full text could not be found; and single group studies.

### Data extraction

Two researchers independently searched the data according to the inclusion and exclusion criteria to select studies for analysis. The selected studies were extracted by author, publication year, country, Institutional Review Board (IRB), fund, number of subjects, study design, intervention characteristics (oil type, application method, application area, control group intervention method, intervention duration, intervention session, intervention time/session, number of measurement time), and outcome variables and then recorded in a coding book created in Microsoft Excel Spreadsheet Software. In case of coding discrepancies, the original articles were reviewed again and final coding values were determined (Table 2).

### Quality assessment

The quality assessment of the selected literatures was independently performed by two researchers using the Joanna Briggs Institute (JBI) checklist. The five articles included in the analysis were all RCTs, with a mean value of 9.40±2.30 using the 13-item JBI Checklist for RCTs tool [10]. Only one article clearly reported " Q7. Were outcome assessors blind to treatment assignment?", and only two articles clearly reported " Q2. Was allocation to treatment groups concealed?", "Q4. Were participants blind to treatment assignment?", and "Q5. Were those delivering the treatment blind to treatment assignment?"

### Statistical analyses

MIX 2.0 Pro (Ver. 2.0.1.6, BiostatXL, 2017) was used to calculate and merge effect sizes for pain, the primary outcome of the studies. For the pooled overall effect of pain, Hedge's g was used as the effect size due to the small number of studies, and a synthesis forest plot was used using a random effects model with reweighting to account for variations in subject characteristics and study-specific heterogeneity. The significance of the effect was determined using 95% confidence interval (CI), p value less than .05, and the weight of each effect size was derived using the inverse of variance [11]. The heterogeneity of the included studies was assessed using Q statistics and Higgin's I2 values [12], and 95% CIs were reported to account for the bias of the heterogeneity I2, which is a point estimate in small meta-analysis studies; an I2 greater than 50% was interpreted as heterogeneity [13]. Meta-regression and exclusion sensitivity analysis were performed to identify factors that determine the heterogeneity of studies on aromatherapy for pain in diabetes. Publication bias was also tested using funnel plot, trim and fill plot, Begg's test, and trim and fill method, correcting for pooled overall effect [14].

## RESULTS

### Characteristics of the included studies

**Figure 1.**
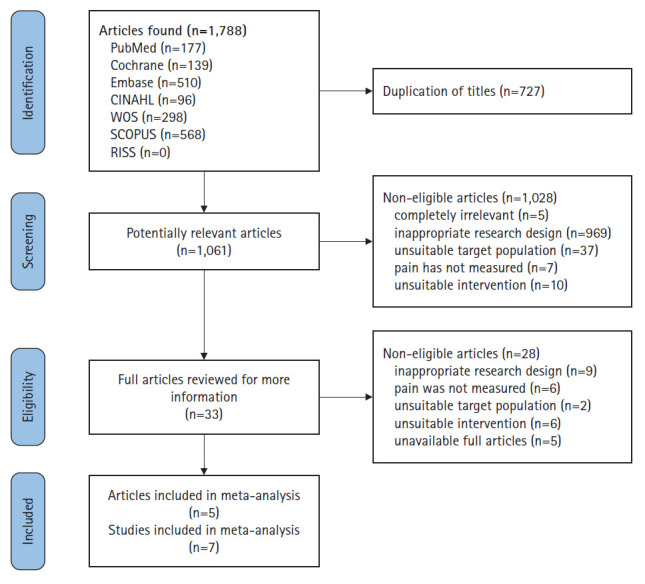
PRISMA flow diagram.

A total of 1,788 articles were retrieved from seven databases according to the search strategy, and 1,061 articles were extracted after excluding duplicates. Based on the inclusion and exclusion criteria, we selected the final five articles (Figure 1). As shown in Table 2, two studies were published after 2021, three were conducted in West Asia, four were reviewed on IRB and one was funded. The study designs were all RCTs, and one studies had fewer than 60 subjects. The characteristics of the interventions were: oil type was lavender oil in two studies and blended oil in two studies; aromatherapy was applied to the feet in two studies, hands and feet in two studies, and other oils were applied in the control group in two studies; intervention duration was 28 days (4 weeks) or more in three studies; intervention sessions were 12 or more in three studies; and intervention time per session was less than 30 minutes in three studies. Three studies measured the dependent variable pre- and post-intervention, two studies measured it mid-intervention, and four studies had a quality score of 9 or higher (Table 2, Appendix 1).

### The effect of aromatherapy on pain among diabetes

**Figure 2.**
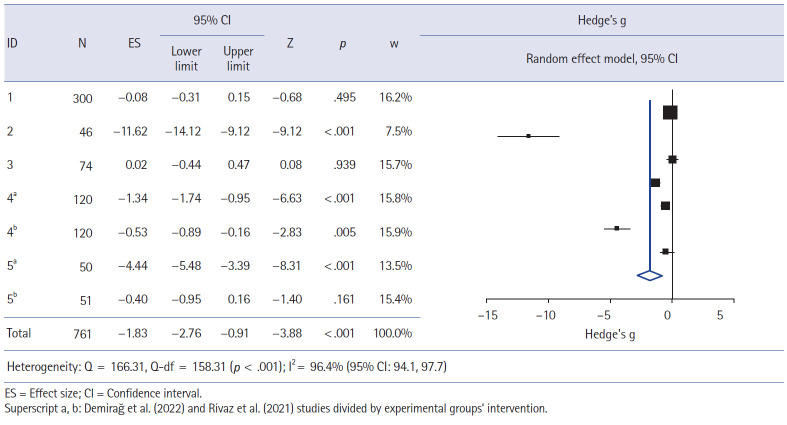
The effect of aromatherapy on pain in diabetes.

The pooled overall effect of pain in diabetes on aromatherapy was Hedge's g = −1.83 (95% CI: −2.76 to −0.91), which was found to be a large effect based on the criteria for interpreting effect sizes outlined by Brydges [15] (Figure 2). The studies were highly heterogeneous, with a Q = 166.31 (Q-df = 158.31, *p* < .001) and a Higgins' I2 of 96.4% in the heterogeneity test. Univariate meta-regression was performed to identify potential influences on the effectiveness of aromatherapy on pain in diabetes and found that West Asian (Z = −6.46, *p* < .001), being reviewed on IRB (Z = −4.92, *p* < .001), and having a fund (Z = −8.75, *p* < .001) were associated with a reduction in pain. In terms of study intervention characteristics, pain reduction was more likely to occur when the participants were younger than 60 (Z = 5.00, *p* < .001), when the type of aromatic oil used was lavender oil (Z = −7.16, *p* < .001), and when the intervention was massage therapy rather than topical application (Z = −5.03, *p* < .001). Fewer hours per session (Z = 4.02, *p* < .001) and more repeated measurements (Z = −5.03, *p* < .001) than two pre- and post-test measurements (Z = −5.03, *p* < .001) were associated with pain reduction. However, the duration of the intervention (Z = −1.88, *p* = .060), number of sessions (Z = 1.11, *p* = .268), and quality of life scores (Z = −1.54, *p* = .123) did not have a significant effect on pain reduction (Table 3). When one study was excluded using an exclusion sensitivity test [16], Hedge's g was −0.99 to −2.36, a large effect size, and the 95% CI (−1.71 to −3.58, −0.27 to −1.17) did not include zero, all of which were statistically significant.

### Publication bias

**Figure 3.**
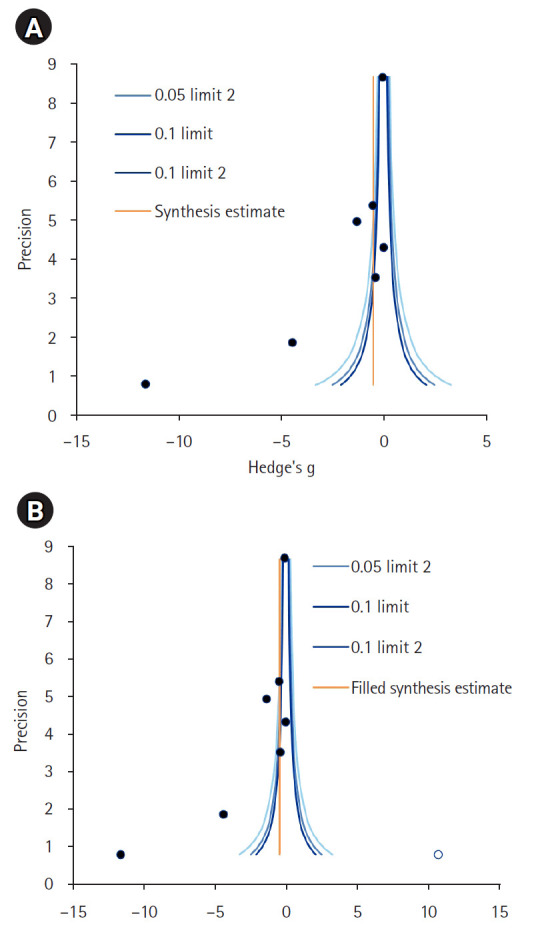
(A) Funnel plot of the effect of aromatherapy on pain. (B) Trim-and-fill plot of the effect of aromatherapy on pain.

To check for publication bias in the study, we performed funnel plot and trim and fill plot analyses and found that the individual effect sizes of the seven studies included in the study (blue circles) were skewed slightly to the left, indicating some publication bias (Figure 3A), and the trim and fill plot showed that one additional study (white circle) should be added (Figure 3B). For further analysis of publication bias, Begg's test was performed, and Tau b = −0.57, ties = 0.00 (Z = −1.80, p = .072), confirming that there was no publication bias. Furthermore, the number of articles that should have been added to the study using the trim and fill method [17] was found to be 1, and the effect size of the 8 corrected studies was −0.49 (95%CI: −0.65, −0.34). The effect size of pain reduction after correction was somewhat smaller than before correction, but it was still statistically significant after correction. In conclusion, the study was free of publication bias (Table 4).

## DISCUSSION

This study aimed to investigate the impact of aromatherapy on pain experienced by diabetes. The overall effect size, as indicated by Hedge's g, was found to be −1.83 (95% CI: −2.76 to −0.91), suggesting a substantial reduction in pain levels. Univariate meta-regression analysis was conducted to explore potential factors influencing the overall effect of aromatherapy on pain in diabetes. Interestingly, compared to patients in South Asia, those in West Asia who received support from IRBs and funding exhibited a lower reduction in pain levels. While interpreting these findings in relation to geographical regions poses challenges, it is inferred that well-designed studies with robust research support from IRBs and funding sources yield more effective results.

Regarding the intervention characteristics of the study, it was observed that cases involving study participants under 60 years of age showed greater efficacy in pain reduction compared to cases where lavender oil was not included. This suggests that lavender oil exhibited a superior pain-reducing effect in diabetic subjects compared to other oil types. This finding aligns with the understanding that each essential oil possesses a unique chemical composition, influencing its aroma, absorption properties, and physiological effects [8]. Lavender oil, specifically derived from *Lavandula angustifolia*, contains linalyl acetate, an ester known for its efficacy in promoting mental stability, muscle relaxation, sterilization, skin rejuvenation, and immune enhancement [7]. Moreover, lavender is renowned for its therapeutic properties, including alleviating headaches, depression, nervous tension, and insomnia, while also exerting sedative, calming, and blood pressure-lowering effects [18].

Previous studies have corroborated the effectiveness of aroma inhalation therapy, demonstrating its ability to reduce headaches and anxiety through complex aroma therapy applications [19], alleviate nausea and vomiting via inhalation of a blend of peppermint and lavender aromas [20], and improve sleep disorders, fatigue, and sleep satisfaction following lavender inhalation therapy [21]. These effects align with the known analgesic, neuroprotective, anti-inflammatory, and muscle relaxant properties of lavender and eucalyptus [22], further validating the consistency of our results with established mechanisms of lavender oil. It is essential to note that different oils may exhibit varying effectiveness depending on the unique disease characteristics of each individual. In particular, our study's findings underscore the efficacy of lavender oil specifically for diabetes. This suggests that exploring the relationship between lavender oil and neuropathy specifically in future research would be valuable, given the high prevalence of neuropathy among diabetes.

Furthermore, our findings indicate that massage therapy was more effective in reducing pain compared to topical application alone. This suggests that the effectiveness of pain relief may vary depending on the method of application. While previous studies have suggested that aromatherapy primarily involves inhalation, our results contradict this notion. Previous research has demonstrated that aromatherapy can be administered through various methods, including inhalation, topical application with or without massage, or in baths. However, our findings indicate that the pain-relieving effect of aromatherapy may be enhanced when combined with massage therapy. This contrasts with the traditional understanding that aroma oils are primarily absorbed through inhalation, where the molecules diffuse to receptors on olfactory sensory neurons, transmitting signals to the brain for interpretation [8]. Nevertheless, our findings align with previous research indicating significant pain reduction with aromatherapy when combined with massage [23]. It is hypothesized that this effect is achieved through the synergistic combination of massage's general effects, such as improved blood circulation and relaxation. In essence, while the aroma oil itself may possess inherent effectiveness, our systematic literature review and meta-analysis reveal that the application of massage enhances its efficacy. While the choice of oil remains crucial, exploring diverse methods of application is imperative to ensure optimal absorption and maximize therapeutic benefits.

Aromatherapy is connected to the brain through the olfactory nerve, which links directly to the limbic system, the most primitive part of the brain [24]. The limbic system governs behaviors related to basic instincts, such as sexual desire, appetite, thirst, and emotional responses [24]. The scent of essential oils stimulates the limbic system via the sense of smell, enhancing memory and immunity, while also reducing tension and stress. Thus, aromatherapy indirectly affects factors that can reduce pain, rather than directly influencing the pain mechanism itself.

On the other hand, it was observed that shorter operating times per session resulted in a greater reduction in pain compared to longer durations. Similarly, a pain reduction effect was noted when the number of measurements was repeated more than twice before and after the intervention. Conversely, factors such as the intervention period, number of intervention sessions, and quality evaluation score did not exhibit a significant effect on pain reduction. This suggests that prolonged operating times per session or intervention periods do not necessarily contribute to pain reduction, indicating that longer durations may not be more effective.

In the context of aromatherapy, it is suggested that aroma oils used in scent inhalation therapy exert an integrated mental and physical effect as volatile odor particles enter through the nose and lungs, ultimately reaching the limbic system of the brain [25]. Therefore, our findings imply that ensuring optimal absorption of the oil, even with shorter exposure durations, may be more effective than solely focusing on prolonging exposure times.

## CONCLUSION

Based on the findings of this study, aromatherapy emerges as an effective treatment modality for alleviating pain in diabetes. Particularly noteworthy is the efficacy observed when employing lavender oil, coupled with massage therapy and shorter application times. These results underscore the importance of considering these factors when contemplating the application of aromatherapy in diabetic care.

## Figures and Tables

**Table 1.** Search Strategy according to the PICO-SD Framework

PICO-SD	Key terms	MeSH	PubMed entry terms	Emtree (Embase)	Text words
P (patients, population, participants, problems)	Diabetes	"Diabetes mellitus"[MeSH]		Diabetes mellitus/	Diabet*
I (intervention or exposure or index test)	Aromatherapy	"Aromatherapy"[MeSH]	Aroma therapy	Aromatherapy/	Aromatherap*
			Therapy, aroma		Aroma therap*
					("oil" OR "aroma*") AND ("massage*" OR "topical*" OR "inhal*")
		"Oils, volatile"[MeSH]	Essential oil	Essential oil/	Essential oil*
			Oil, essential		Volatile oil*
			Oil, volatile		Aromatic oil*
			Oils, essential		
			Volatile oil		
				Fragrance/	Fragran*
					Scent*
		"Plant extracts"[MeSH]	Herbal medicines	Plant extract/	("plant*" OR "herb*" OR "lavend*" OR "rosemar*" OR "orange" OR "citrus") AND ("massage*" OR "topical*" OR "inhal*")
			Plant extract		
		"Plants, medicinal"[MeSH]	Healing plants	Medicinal plant/	
			Herbs, medicinal		
			Medicinal herbs		
			Medicinal plants		
			Pharmaceutical plants		
O (outcomes, effects)	Pain	"Pain"[MeSH]	Ache		Pain*
			Pain, burning		Ache*
			Pain, crushing		
			Pain, migratory		
			Pain, radiating		
			Pain, splitting		
			Suffering, physical		
		"Neuralgia"[MeSH]	Nerve pain		Neuralgi*
			Neuralgia, atypical		Neuropath*
			Neuralgia, iliohypogastric nerve		
			Neuralgia, ilioinguinal		
			Neuralgia, perineal		
			Neuralgia, stump		
			Neuralgia, supraorbital		
			Neuralgia, vidian		
			Neurodynia		
			Neuropathic pain		
			Paroxysmal nerve pain		
		"Diabetic neuropathies"[MeSH]	Asymmetric diabetic proximal motor neuropathy		Amyotroph*
			Diabetic amyotrophy		Polyneuropath*
			Diabetic asymmetric polyneuropathy		Mononeuropath*
			Diabetic autonomic neuropathy		
			Diabetic mononeuropathy		
			Diabetic mononeuropathy simplex		
			Diabetic neuralgia		
			Diabetic neuropathy, painful		
			Diabetic polyneuropathy		
			Mononeuropathy, diabetic		
			Neuralgia, diabetic		
			Symmetric diabetic proximal motor neuropathy		
		"Nociceptive pain"[MeSH]	Somatic pain		Nocicept*
			Tissue pain		
		"Myofascial pain syndromes"[MeSH]	Myofascial trigger point pain		Myofascial
			Trigger point pain, myofascial		
		"Pain measurement"[MeSH]	Analgesia tests		Analog scale
			Analog pain scale		Analogue scale
			Analogue pain scale		
			Assessment, pain		
			Formalin test		
			McGill pain questionnaire		
			McGill pain scale		
			Nociception tests		
			Pain assessment		
			Pain intensity		
			Pain severity		
			Tourniquet pain test		
			Visual analog pain scale		
			Visual analogue pain scale		
		"Analgesia"[MeSH]			Analges*
		"Pain management"[MeSH]			
		"Palliative care"[MeSH]	Palliative supportive care		Palliative
			Palliative surgery		
			Palliative therapy		
			Palliative treatment		
			Surgery, palliative		
			Therapy, palliative		
Study design	RCT, quasi-experimental				
Restrictions	English, korean / humans (adult: 19+ years), (young adult: 19-24 years) male, female / 1900.01.01 - 2023.12.31				

PICO-SD = Population, intervention, comparison, outcome, study design; RCT = Randomized controlled trial.

**Table 2.** Characteristics of the Included Studies

Study	Author (yr)	Country	IRB	Fund	Participants	Research design	Oil type	Experimental G intervention (application method)	Experimental G intervention (application area)	Control G intervention	Intervention duration	Intervention session	Time/session (min)	Measurement time	Outcome variables (measurement tool)	Quality score
[A1]	Musharraf et al. (2017)	Pakistan	No	No	300 patients with painful diabetic neuropathy (E: 150, C: 150)	RCT	Turpentine oil	Topical application	Feet	Capsaicin (Capcidol® cream)	1 day	1	30	2	- Neuropathic pain (VAS)	7
[A2]	Metin et al. (2017)	Turkey	Yes	Yes	46 patients with painful diabetic neuropathy (E: 21, C: 25)	RCT	Blend of five essential oils	Massage	Feet, hands	Routine care	28 days (4 wks)	12	30	3	- Neuropathic pain (DN4)	8
															- Pain (VAS)	
															- Quality of life (NePIQoL)	
[A3]	Motilal & Maharaj (2013)	India	Yes	No	74 patients with diabetes (E: 37, C: 37)	RCT	Blended oils	Topical application	Feet, hands	Placebo	28 days (4 wks)	84	10	2	- Pain (BPI-DPN, NPSI)	13
															- Compliance	
[A4]	Demirağ et al. (2022)	Turkey	Yes	No	180 patients with diabetes (E1: 60, E2: 60, C: 60)	RCT	E1: lavender oil, E2: placebo (distiled water)	Topical application	Arms	No intervention	1 day	1	5	2	- Insulin injection pain (VCS, VAS)	10
															- BP, RR, PR, SPO_2_	
															- Blood glucose	
[A5]	Rivaz et al. (2021)	Iran	Yes	No	75 patients with diabetic neuropathy (E1: 26, E2: 25, C: 24)	RCT	E1: lavender oil, E2: sunflower oil	Massage	Feet	No intervention	28 days (4 wks)	28	10	3	- Neuropathic pain (VAS, DN4)	9
															- Quality of life (SF-36)	
Total score (M ± SD)																9.40 ± 2.30

IRB = Institutional review board; G = Group; E = Experimental group; C = Control group; RCT = Randomized controlled trial; VAS = Visual analogue scale score; DN4 = Douleur neuropathique questionnaire, NePIQoL = Neuropathic pain impact on quality of life questionnaire; BPI-DPN = Brief pain inventory for diabetic painful neuropathy; NPSI = Neuropathic pain symptom inventory; VCS = Verbal category scale, BP = Blood pressure; RR = Respiratory rate; PR = Pulse rate; SPO_2_ = Oxygen saturation level; SF-36 = Short-form health survey; M = mean; SD = Standard deviation.

**Table 3.** Meta-regression Analysis for Pain

Covariates (Ref.)	Estimate	SE	95% CI		Z	*p*
			Lower limit	Upper limit		
Country (South Asia)	−1.02	0.16	−1.33	−0.71	−6.46	< .001
IRB (No)	−0.77	0.16	−1.08	−0.46	−4.92	< .001
Fund (No)	−11.16	1.28	−13.66	−8.66	−8.75	< .001
Participants (< 60)	1.30	0.26	0.79	1.81	5.00	< .001
Aromatherapy oil type (Without lavender oil)	−1.49	0.21	−1.90	−1.08	−7.16	< .001
Aromatherapy method (Topical application)	−1.30	0.26	−1.81	−0.79	−5.03	< .001
Aromatherapy application area (Without feet)	−0.36	0.19	−0.73	0.01	−1.88	.060
Intervention duration	−0.01	0.01	−0.03	0.00	−1.88	.060
Intervention session	0.00	0.00	0.00	0.01	1.11	.268
Intervention time/session	0.03	0.01	0.01	0.04	4.02	< .001
Number of outcome measurement	−1.30	0.26	−1.81	−0.79	−5.03	< .001
Quality score	−0.06	0.04	−0.14	0.02	−1.54	.123

Ref. = Reference; SE = Standard error; CI = Confidence interval; IRB = Institutional review board.

**Table 4.** Publication Bias Test of the Effect of Aromatherapy on Pain

Begg's test	Tau b	K	S (P−Q)	ties	Z	*p*
Standard	−0.62	7	−13.00	0.00	−1.95	.051
Corrected	−0.57	7	−13.00	0.00	−1.80	.072
Trim-and-fill method	K	Hedge’s g	95% CI		Z	*p*
			Lower limit	Upper limit		
Original	7	−1.83	−2.76	−0.91	−3.88	<.001
Corrected	8	−0.49	−0.65	−0.34	−6.28	<.001

Begg's test for rank correlation.

CI = Confidence interval; K = Number of analysis set.

## Data Availability

The data that support the findings of this study are available from the corresponding author upon reasonable request.

## Appendix 1. Studies included in the systematic review and meta-analysis

A1. Musharraf MU, Ahmad Z, Yaqub Z. Comparison of topical capsaicin and topical turpentine oil for treatment of painful diabetic neuropathy. Journal of Ayub Medical College Abbottabad. 2017;29(3):384-387.

A2. Metin ZG, ArikanDonmez A, Izgu N, Ozdemir L, Arslan IE. Aromatherapy massage for neuropathic pain and quality of life in diabetic patients. Journal of Nursing Scholarship. 2017;49(4):379-388. https://doi.org/10.1111/jnu.12300

A3. Motilal S, Maharaj RG. Nutmeg extracts for painful diabetic neuropathy: a randomized, double-blind, controlled study. The Journal of Alternative and Complementary Medicine. 2013;19(4):347-352. https://doi.org/10.1089/acm.2012.0016

A4. Demirağ H, Hintistan S, Bulut E. The effect of topically administered lavender aromatherapy on the pain of insulin injection in diabetic patients: a double-blind randomized controlled clinical trial. Turkish Journal of Medical Science. 2022;52(6):1845-1853. https://doi.org/10.55730/1300-0144.5531

A5. Rivaz M, Rahpeima M, Khademian Z, Dabbaghmanesh MH. The effects of aromatherapy massage with lavender essential oil on neuropathic pain and quality of life in diabetic patients: a randomized clinical trial. Complementary Therapies in Clinical Practice. 2021;44:101430. https://doi.org/10.1016/j.ctcp.2021.101430
